# Disease Resistance Mechanisms in Plants

**DOI:** 10.3390/genes9070339

**Published:** 2018-07-04

**Authors:** Ethan J. Andersen, Shaukat Ali, Emmanuel Byamukama, Yang Yen, Madhav P. Nepal

**Affiliations:** 1 Department of Biology and Microbiology, South Dakota State University, Brookings, 57007 SD, USA; ethan.andersen@sdstate.edu (E.A.); yang.yen@sdstate.edu (Y.Y.); 2 Department of Agronomy, Horticulture, and Plant Science, South Dakota State University, Brookings, 57007 SD, USA; shaukat.ali@sdstate.edu (S.A.); emmanuel.byamukama@sdstate.edu (E.B.)

**Keywords:** coevolution, defense response, disease resistance, nucleotide-binding leucine-rich repeat, plant immunity, plant-pest interaction, pathogenesis, pattern recognition receptor, phytopathology, resistance gene

## Abstract

Plants have developed a complex defense system against diverse pests and pathogens. Once pathogens overcome mechanical barriers to infection, plant receptors initiate signaling pathways driving the expression of defense response genes. Plant immune systems rely on their ability to recognize enemy molecules, carry out signal transduction, and respond defensively through pathways involving many genes and their products. Pathogens actively attempt to evade and interfere with response pathways, selecting for a decentralized, multicomponent immune system. Recent advances in molecular techniques have greatly expanded our understanding of plant immunity, largely driven by potential application to agricultural systems. Here, we review the major plant immune system components, state of the art knowledge, and future direction of research on plant–pathogen interactions. In our review, we will discuss how the decentralization of plant immune systems have provided both increased evolutionary opportunity for pathogen resistance, as well as additional mechanisms for pathogen inhibition of such defense responses. We conclude that the rapid advances in bioinformatics and molecular biology are driving an explosion of information that will advance agricultural production and illustrate how complex molecular interactions evolve.

## 1. Introduction

### 1.1. Plant Disease Resistance Has Emerged as a Complex, Multicomponent System

The agricultural revolution of approximately 10,000 years ago intensified humanity’s relationship with plants. Since survival largely depended on yield, early farmers would have selected plants based on multiple factors, including their ability to resist disease. Multiple plant diseases recorded in ancient times were often attributed to supernatural causes, but phytopathological observations can be traced back to the third and fourth centuries B.C. in the writings of Aristotle’s student Theophrastus [1]. Microbiology during the enlightenment enabled systematic classification of pathogenic organisms, newly visible under compound microscopes. Early experiments in the 19th century demonstrated the efficacy of fungicides, such as the Bordeaux mixture of copper sulfate and calcium oxide [2]. Also in the 19th century, pathogenic microbes were demonstrated to be the causal agents of plant diseases by Heinrich Anton de Bary [3]. Understanding of heritability and genetics, developed in the early 20th century, allowed researchers to identify sources of heritable resistance, called resistance genes (R genes) [4,5]. R genes were further described by Harold Henry Flor’s groundbreaking gene-for-gene model [6], correlated with avirulence genes present in the pathogen that create an incompatible response. Mechanisms for resistance utilizing R genes were then elucidated following advances in chemistry and molecular biology later in the 20th century [7,8]. The advances in molecular techniques and genomics of the early 21st century drove the discovery of numerous classes of genes that encode regulators of disease resistance and susceptibility [9]. R genes were found to be only one set of participants in a web of interacting factors. Recent molecular research has revealed that plant resistance relies on a complex regulatory system that controls plant defense responses, greatly building upon the simple structure of H. H. Flor’s gene-for-gene model. Plant immune system components participate in pathogen detection, signal transduction, or defense response. Selection pressures drive the evolution of plants with complex detection systems and pathogens with sophisticated evasion techniques, as outlined in the zig-zag model [10]. This review focuses on these components, and how they are involved in immunity. We do not aim to completely exhaust every aspect of each component, as whole reviews are dedicated to a single class of one factor. Instead, we hope to present the conceptual framework of phytopathogen resistance, as supported by examples. We will first briefly review which components are involved in this plant immune system, and how pathogens have evolved to subvert defenses.

### 1.2. Plant Defense Relies on Detection and Response

**Figure 1:**
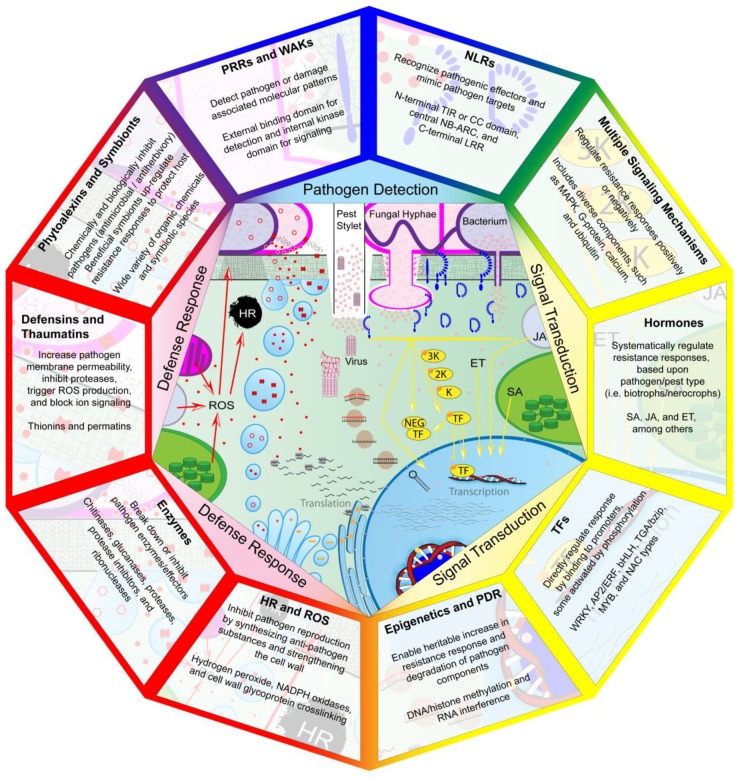
Components of plant disease resistance mechanisms involved in pathogen detection, signal transduction, and defense response (detection in the upper center and progressing around clockwise, ending in defense response in the upper left). Pathogenic elicitors (cell components or effectors) produced by bacteria, fungi, insects, nematodes, or viruses trigger plant receptors to initiate signaling cascades. Activated receptors (blue) then initiate one of many signal transduction pathways or directly act as transcription factors (TFs). Signal transduction pathways (yellow) include mitogen-activated protein kinase (MAPK) cascades, calcium ion signaling, hormone production, TF activity, and epigenetic regulation. These factors trigger the expression of genes associated with defense responses, such as those regulating the production of reactive oxygen species (ROS), antimicrobial enzymes, defensins, and phytoalexins. These defense-related compounds (red) actively inhibit pathogen reproduction or make further infection more difficult. Breakdown of pathogenic cell components by defense compounds leads to further release of receptor-triggering elicitors, increasing the resistance response. Multiple organelles are involved in defense response, including chloroplasts and peroxisomes for hormone production as well as the nucleus, endoplasmic reticulum, and Golgi apparatus for antimicrobial protein production. PRR: Pattern recognition receptors; WAK: wall-associated kinases; NLR: nucleotide-binding domains and leucine-rich repeats; PDR: pathogen-derived resistance; HR: hypersensitive response; TIR: N-terminal Toll/interleukin-1 receptor-like; CC: coiled-coil; SA: salicylic acid; JA: jasmonic acid; ET: ethylene.

Investigation into the molecular basis of pathogen resistance reveals a suite of cellular receptors that performs direct detection of pathogenic molecules. Pattern recognition receptors (PRRs) within the cell membrane detect pathogen-associated molecular patterns (PAMPs) and wall-associated kinases (WAKs) detect damage-associated molecular patterns (DAMPs) that result from cellular damage during infection [11,12]. Receptors with nucleotide-binding domains and leucine-rich repeats (NLRs) detect effectors that pathogens use to facilitate infection [13]. PRRs, WAKs, and NLRs initiate one of many signaling cascades that have yet to be completely elucidated. Mitogen-activated protein kinases (MAPKs), G-proteins, ubiquitin, calcium, hormones, transcription factors (TFs), and epigenetic modifications regulate the expression of pathogenesis-related (PR) genes [14,15,16,17,18]. This leads to various responses that prevent further infection: hypersensitive response (HR), production of reactive oxygen species (ROS), cell wall modification, closure of stomata, or the production of various anti-pest proteins and compounds (e.g., chitinases, protease inhibitors, defensins, and phytoalexins) [19,20]. As now understood from molecular techniques, pathogen resistance in plants involves various organelles and classes of both proteins and non-protein compounds, each of which are required to regulate defense response (see Figure 1). Factors in each of these roles affect various other signaling systems, such as growth and abiotic stress response. An improved understanding of plant–pathogen interaction requires that we fully describe these molecular interactions that take place when a compatible pathogen interacts with plant tissue. First, however, we must briefly describe the pathogens that elicit these responses, and how their evolution has led to the complex immune system that plants possess.

### 1.3. Pathogen Virulence Relies on Attack and Evasion

Diverse species and races of pathogens drive plant populations to evolve a highly varied set of immune receptors and modes of response [8,21,22]. Pathogens respond by evolving mechanisms to evade host perception or negate defense responses [10]. Pathogens often subvert PAMP-triggered immunity (PTI) through effectors, which can be secreted through various systems. The bacterium *Xanthomonas translucens*, for example, secretes 20–40 effectors through a type III secretion system, resembling a tube that transfers effectors into wheat cytoplasm [23]. Other members of the *Xanthomonas* genus utilize a type VI secretion system, which transfers effectors extracellularly [24]. Pathogens can also increase virulence by transferring effector genes from one species to another, called horizontal gene transfer. Such an event has been proposed to describe the transfer of the necrosis-inducing *ToxA* gene from *Parastagonospora nodorum* to *Pyrenophora tritici-repentis*, which causes the disease tan spot in wheat [25]. Plant effector-triggered immunity (ETI) detects these effectors and initiates a defense response. Such responses must also be varied as pathogens differ in how they extract nutrients: biotrophs from living tissue and necrotrophs from dead tissue. HR intended to inhibit biotrophs only facilitates necrotroph infection [26]. Hemi-biotrophs trick the plant by switching from biotrophy to necrotrophy [27], producing effectors in waves that differ by tissue and infection stage [28]. The complex system leading to pathogen resistance, described in Figure 1, has been subverted at various levels, with pathogens evolving effectors that compromise many of the various signaling steps. As we review next, the first stage of pathogen detection relies upon the recognition of molecules unique to pathogens or resulting from pathogen infection.

## 2. Pathogen Detection

### 2.1. PRRs and WAKs Detect PAMPs and DAMPs

PRRs are able to recognize a wide array of microbial components, including fungal carbohydrates [29], bacterial proteins [30], and viral nucleic acids [31]. These receptors often possess leucine-rich repeats (LRRs) that bind to extracellular ligands, transmembrane domains necessary for their localization in the plasma membrane, and cytoplasmic kinase domains for signal transduction through phosphorylation [11]. LRRs are highly divergent, associated with their ability to bind to diverse elicitors. Many PRRs rely on the regulatory protein brassinosteroid insensitive 1-associated receptor kinase 1 (BAK1) and other somatic embryogenesis receptor-like kinases (SERKs) [32,33]. Extensive signaling is not always initiated, as some PRRs, upon activation, release kinase domains that travel to the nucleus to trigger transcriptional reprogramming [34]. Molecules detected by PRRs are diverse: bacterial (flagellin, elongation factor EF-Tu, and peptidoglycan) [35,36,37], fungal (chitin, xylanase) [38,39], oomycete (β-glucan and elicitins) [40,41], viral (double stranded RNA) [31], and insect (aphid-derived elicitors) [33]. Although many of these studies were conducted to elucidate specific molecular interactions in *Arabidopsis*, wheat PRRs TaLRK10, TaRLP1.1, and TaRLK-R1-3 have been associated with resistance to rust (fungi of the genus *Puccinia*) via detection of fungal PAMPs [42,43,44].

Unlike the PRRs that detect non-self pathogen molecules upon infection, other receptors perceive damage by recognizing cellular components that have been disrupted by pathogenic enzymes. This has been shown in *Arabidopsis* with WAK1 perception of oligogalacturonides [45] or DORN1/LecRK-I.9 perception of extracellular ATP [46]. WAKs possess an N-terminal, extracellular galacturonan-binding domain that interacts with pectins in the cell wall, and cytoplasmic kinase domains, similar to the structure of PRRs. WAK1 and WAK2 perceive oligogalacturonic acid, resulting from plant cell wall pectin degradation by fungal enzymes [45]. Plant lectins are able to recognize carbohydrates that originate directly from pathogens or from damage incurred during infection [47]. Many PAMPs and DAMPs contain carbohydrates (i.e., lipopolysaccharides, peptidoglycans, oligogalacturonides, and cellulose) and are recognized by PRRs/WAKs with lectin domains, such as lectin receptor kinases [47]. Plants detect many extracellular molecules that indicate pathogen infection [48], such as extracellular DNA, ATP, and NAD(P). Pathogens have evolved to interfere in the detection of PAMPs and reduce the efficacy of PTI. *Cladosporium fulvum* and *Magnaporthe oryzae* produce chitin-binding proteins in order to prevent plant perception (i.e., Avr4 and Slp1, respectively) [49,50,51]. Pathogens also produce effectors to thwart many aspects of plant immunity, which plants have developed ways to overcome, as outlined in the zig-zag model [10]. In order to recognize these infection-facilitating pathogen effectors, plants utilize another, more varied class of proteins.

### 2.2. NLRs Detect Pathogen Effectors

NLRs, also known as R genes, are among the fastest evolving gene families. Their products, upon detection of pathogenic effectors, undergo a conformational shift from a condensed, ADP-bound state to an open ATP-bound state with exposed N-terminal domains for the initiation of downstream signaling [52,53,54]. N-terminal Toll/interleukin-1 receptor-like (TIR) or coiled-coil (CC) domains precede an evolutionarily conserved domain with a Nucleotide-Binding site present in Apoptotic protease-activating factor, R proteins, and *Caenorhabditis elegans* death-4 protein (NB-ARC), followed by and a highly variable LRR [52]. CC-NBS-LRR genes (CNL genes) are found in both monocots and dicots, but TIR-NBS-LRR genes (TNL genes) are restricted only to the latter [55]. Similar to PRRs, variability in the LRR provides these receptors with the ability to recognize various effector structures. Unlike PRRs, NLRs are generally located in the cytoplasm and possess LRRs at the C-terminal end. The NB-ARC contains many conserved motifs: P-loop/Walker-A, resistance nucleotide-binding site A (RNBS-A), Kinase-2/Walker-B, RNBS-B, RNBS-C, GLPL, RNBS-D, and MHDV, GLPL and MHDV named after the conserved amino acids present. However, not all motifs are required for function, exemplified by the rice Pb1 CNL protein, which lacks a P-loop [56]. Wu et al. (2016 and 2017) have shown that NLRs operate in networks, differentiating sensor and helper NLRs, along with NLRs required for cell death (NRCs) [57,58]. NLRs have emerged as complex receptors that can detect a variety of changes, both through non-self and modified-self recognition [59]. NLR interactions are not always advantageous, as mismatching of NLRs in hybrids can result in autoimmunity [60]. NB-ARCs similar to those found in NLRs are found in many different species, from the bacterial *Streptomyces coelicolor* genome [61] to those of nematode [62] and human [63], the latter two also involved in programmed cell death. Many NLR genes are located in extra-pericentromeric clusters [64], which experience high rates of chromosomal recombination. These genes evolve quickly through duplications, chromosomal rearrangements, and unequal crossing over [65]. Transposable elements also play a role in the evolution of regulatory sequences, like promoters [66,67]. Translocation of NLR genes to unlinked loci increases the likelihood of functional diversification [57]. Similar to PRRs, many effector–NLR interactions have been elucidated in *Arabidopsis* [68]. NLRs become activated by direct interaction with pathogen effectors [69] or by detecting modification of the effector’s target protein [21,70], modification of a target-mimicking decoy protein [71], or modification of the NLR itself [72,73,74]. One of the most well-studied NLR–effector interactions involves the NLRs RPM1 and RPS2, which perceive the targeting of resistance negative regulator RIN4 by *Pseudomonas syringae* effectors AvrRpt2 and AvrRpm1 [75]. Unlike other NLRs, RPS2 and RPM1 are located within the plasma membrane, since their guardee, RIN4, is also associated with the plasma membrane [76]. After activation, RPM1 associates with the promoter-binding AtTIP49a negative regulator, to upregulate resistance responses [77].

NLRs have diverse structures that may include integrated decoy domains that exist as targets for effectors, such as TF sequences that effectors regularly target. *Arabidopsis* resistance to *Ralstonia solanacearum* 1 (RRS1) has a WRKY TF domain at the C-terminal end, to directly bind to promoter sequences [78]. The *Ralstonia solanacearum* effector PopP2 modifies this WRKY domain, which triggers activation of the NLR [79,80]. NLRs are also able to dimerize, such as CNL proteins RGA5 and RGA4 in rice, in which RGA5 directly binds to *M. oryzae* effectors Avr-Pia and Avr1-Co39 [81,82]. Much research is still necessary to elucidate how NLRs self-associate or dimerize, specifically genes with economic importance, such as wheat stem rust resistance genes [83]. Similar to PRR reliance on BAK1, NLRs rely on other proteins to transmit signals. CNLs and TNLs associate with non-race-specific disease resistance 1 (NDR1) and enhanced disease susceptibility 1 (EDS1) proteins, respectively [84,85,86]. NLRs are also able to localize to specific areas of the cell, such as the endosomes or nucleus. Potato CNL protein R3a, when triggered by *Phytophthora infestans* effector Avr3a^KI^, moves to endosomes, where it recruits additional effectors [87]. Barley CNL MLA proteins accumulate in the nucleus to interfere with WRKY TFs to downregulate immunity [88]. Plants also use exocytosis to move immune receptors to the plasma membrane and secrete antimicrobial substances. As a way to benefit from disruption of antimicrobial compound production, some pathogen effectors interfere with protease secretion [89], vesicular trafficking via proteasome degradation [90], and endocytosis [91]. *P. nodorum* and *P. tritici-repentis* effectors SnTox1 and PtrToxA utilize susceptibility genes *Snn1* and *Tsn1*, which encode WAK and NLR proteins, respectively [92,93,94,95,96]. This hijacking of immune components allows necrotrophic pathogens to trigger HR through reactive oxygen species (ROS) accumulation [97]. PRRs, WAKs, and NLRs rely on complex signaling mechanisms to initiate defense responses. MAPKs, hormones, TFs, and other components play major roles in this signal transduction.

## 3. Signal Transduction

### 3.1. Resistance Involves Multiple Signaling Mechanisms

Receptors activate signaling mechanisms that are common to many cellular processes, including MAPKs, G-proteins, ubiquitin, and calcium fluctuations. In the general model of MAPK signaling, membrane-bound Ras proteins facilitate the conversion of GTP to GDP, phosphorylating MAPKKK (Raf) proteins, which then phosphorylate MAPKK (MEK) proteins, leading to the phosphorylation of MAPK (ERK) proteins [14]. The involvement of MAPK in many cellular processes has led to the identification of MAPK genes in *Arabidopsis*, which contains 60 MAPKKKs, 10 MAPKKs, and 20 MAPKs [98]. Initiated by bacterial flagellin and elongation factor interaction, PRRs FLS2 and EFR dimerize with BAK1 and trigger MAPK signaling [99,100]. Pathogen pectin degradation detected by WAK1 and WAK2 also initiates a MAPK cascade [45,101,102]. Studies in tomato show MAPK genes involved in signal transduction of NLR perception as well [103,104,105]. Defense responses can also be downregulated by MAPK signaling [106], and pathogens have developed effectors that interfere with MAPK signaling to suppress resistance responses [107]. Similarly, the heterotrimeric G-protein and G-protein-coupled receptor (GPCR) system has been heavily studied due to its involvement in numerous cellular processes. Extracellular ligands bind to the transmembrane GPCR, causing the exchange of GDP for GTP in the α subunit of the G-protein complex, causing a dissociation of the α subunit from the β–γ subunit complex, initiating further signaling [108]. Hydrolysis of GTP by the α subunit then causes the subunits to re-associate. Metazoan systems make more use of G-protein signaling [109,110,111], but G-proteins possess roles in HR and stomatal closure [15]. Ubiquitination and subsequent protein degradation by the proteasome also has activity in many signaling systems, including defense. Components are regulated positively by the repression of their degradation or negatively by targeted degradation [17]. Pathogens have evolved effectors to interfere with the ubiquitin proteasome system in an attempt to disrupt this signaling and facilitate infection [17]. Small ubiquitin-like modifiers (SUMOs) are also utilized by plants to regulate response, and pathogens disrupt this signaling as well [112].

Receptors triggering fluctuations in calcium ions (Ca^2+^) act as signaling mechanisms to trigger responses to symbiotic or pathogenic microbes [16,113]. Calmodulin (CaM), calcium-dependent protein kinases (CDPKs), and calcineurin B-like proteins detect calcium to activate diverse families of TFs, including calmodulin-binding transcription activators (CAMTAs) [113,114]. CaM is involved in ROS production through MAPK cascade initiation [115]. Calcium signaling controls hormone activation and the expression of NDR1 and EDS1 proteins [113]. CDPKs move to the nucleus to phosphorylate WRKY TFs involved in RPS2 and RPM1 ETI [116]. This molecular signal can be transmitted through hormones that have roles in many different stress and developmental responses [14]. Similar to calcium signaling, fluctuations in hormones drive differential expression of defense response genes. While sustained MAPK activity during ETI allows for less reliance on hormonal regulation, transient MAPK activity during PTI depends heavily upon hormonal signaling [117].

### 3.2. Plant Hormones Systematically Initiate and Repress Resistance

Hormones operating downstream of pathogen detection provide another layer of regulation and take many forms: salicylic acid (SA), jasmonic acid (JA), ethylene (ET), abscisic acid (ABA), nitric oxide (NO), cytokinins (CK), gibberellin (GA), auxin, and brassinosteroids (BR). Along with affecting a multitude of developmental and response functions, including crosstalk with other hormones, SA plays a central role in local and systemic resistance responses to biotrophic and hemi-biotrophic pathogens [118]. SA and MAPK cascades can act upstream of each other, with some cascades triggering SA activity, or SA triggering MAPK cascades [118]. Signaling is transferred from receptors to SA through NDR1 for CNL receptors and a combination of EDS1 and phytoalexin deficient 4 (PAD4) for TNL receptors [86,119,120]. Through a complex web of interactions, SA transfers the signal that a pathogen is present through the action of TFs to induce the expression of defense-related genes. After initiated by signaling, SA leads to the reduction of disulfide bonds in the oligomer protein nonexpressor of pathogen resistance gene 1 (NPR1) by thioredoxins, allowing its constituent monomers to pass from the cytosol into the nucleus, bind to the TF TGA (binding site: TGACG), and upregulate genes associated with resistance [18,121]. Taking advantage of this system, the *Cochliobolus victoriae* pathogenic effector, victorin, targets the thioredoxin TRX-h5, involved in the monomerization of NPR1, and triggers cell death through the action of *Arabidopsis* susceptibility protein LOV1 [122].

JA and ET play key roles in the plant’s response to necrotrophic pathogens [18,123,124,125] and herbivorous insects [126,127]. JA and ET upregulate the emission of volatile compounds in response to caterpillar herbivory, initiated by caterpillar oral secretions [128]. In order to subvert plant response, insect gut microbes can reduce the JA-mediated defense in plants [129]. Perception of bacterial flagellin enhances the production of ET as a signaling mechanism [130]. Without ET present, TF ethylene insensitive 3 (EIN3) is degraded by F-box protein-mediated ubiquitination and proteasome activity. ET inactivates receptors and the constitutive triple response1 (CTR1) protein, which stops the repression of EIN2 and EIN3, and allows upregulation of ET signaling, expression of defense genes, and necrotroph resistance [18,131]. This is also a target of pathogen interference, as the XopD effector of tomato pathogen *Xanthomonas euvesicatoria* desumoylates the TF SIERF4, to interfere with hormone signaling, specifically suppressing ET production and resistance [132]. Functioning of the hormones ABA, NO, auxin, CK, GA, and BR in immunity and development shows that defense and growth are closely linked [133], often inversely related. ABA is involved in various plant stresses [134], including the repression and promotion of resistance responses during presence and absence of abiotic stress, respectively [135]. Closure of stomata involves ABA signaling to regulate water loss, gas exchange, and pathogen access to tissue [136,137]. GA and ascorbic acid (AA) deficiencies lead to enhanced resistance [138,139]. Since hormones are broadly defined as systemic regulators, peptides can also function as plant hormones. Recently, the small peptide hormone systemin has been shown to be involved in system herbivory response, leading to changes in gene expression, specifically of neighboring plants that are not exposed to the biotic stress [140]. This indicates that plant hormones can trigger communication between individual plants to increase defense responses. Having discussed some of the major signaling factors, we now shift our attention to the proteins that directly alter transcription in the nucleus.

### 3.3. TFs Play an Essential Role in Transcriptional Reprogramming

Transcriptional reprogramming through TF activity plays roles at several levels of resistance: (1) basal expression of resistance components (e.g., receptors, kinases, response suppression proteins), (2) direct TF activity of activated receptor proteins, and (3) TFs activated downstream of receptor initiation (i.e., MAPK cascade leading to TF activation via phosphorylation [141]). Plant TFs are diverse in comparison to metazoan systems: homeodomain, MADS box, C_2_H_2_ zinc finger, AP2/ERF, bHLH, TGA/bZIP, MYB, NAC, and WRKY; the latter six families are especially involved in defense [131]. AP2/ERF defense-related TFs (binding to GCC boxes: GCCGCC) are associated with ethylene regulation and involved in positive regulation of rice resistance to *Chilo suppressalis* [142] and *Arabidopsis* resistance to *Botrytis cinerea* [143,144]. In the latter example, ethylene response factor 6 (ERF6) activates plant defensin 1.1 and 1.2 after MPK3/MPK6 phosphorylation [145]. An MYC2 TF, NaMYC2 regulates the production of nicotine, an anti-herbivory secondary metabolite from *Nicotiana attenuata* [146]. The chitin-triggered rice bZIP TF OsTGAP1 upregulates antimicrobial compound synthesis [147]. Abiotic stresses can also impact resistance signaling, like cold, drought, or wounding [148,149]. bHLH TF MYC2 works antagonistic to ethylene response factor 1 (ERF1) by downregulating resistance and upregulating wounding responses [150]. Aside from kinase phosphorylation, TFs can also be activated by modifications, such as proteolysis in no apical meristem (NAM)-*Arabidopsis* transcription activation factor (ATAF)-cup-shaped cotyledon (CUC2) (NAC) TF NTL6 [148]. TFs can also target specific aspects of infection, like barley HvNAC6 upregulating genes involved in resistance to *Blumeria graminis* penetration [151]. In addition to these classes of TFs, WRKYs appear to be involved in many of the thoroughly studied resistance responses.

WRKY TFs bind to W-box regions (TTGACC/T) and possess conserved WRKYGQK amino acid residues along with zinc finger domains [152]. In addition to their involvement in abiotic stresses, like drought and salt tolerance, WRKY TFs positively and negatively regulate the expression of genes associated with defense, including response to viruses [153]. WRKY TFs are classified into groups I, IIa–e, and III, with analysis of ancestral species (i.e., algae) leading to potential phylogenetic relationships [152]. *Arabidopsis* resistance to *B. cinerea* involves phosphorylation of WRKY33 by MPK3/MPK6, followed by WRKY33 binding to its own promoter and the promoters of components necessary for the synthesis of ethylene [154] and antimicrobial compounds (via phytoalexin deficient 3) [155]. By contrast, barley HvWRKY1 and HvWRKY2 repress PTI, disrupted by CNL protein mildew locus A10 (MLA10) after detection of the *B. graminis* effector AVR_A10_ [88]. Barley CNL protein MLA1 interacts with WRKY1 and MYB6 TFs, the latter upregulating *B. graminis* resistance [156]. Rice WRKY45 positively regulates blast resistance when activated by the CNL protein Pb1 [157]. Receptor TF activity is shown in resistance to *X. oryzae*, where rice PRR/RLK Xa21 is cleaved and sends a kinase domain to the nucleus that binds to the negative regulator OsWRKY62 [34,158]. The allelic variants encoding proteins OsWRKY45-1 and OsWRKY45-2 both positively regulate resistance to *Magnaporthe grisea*, but downregulate and upregulate resistance to *X. oryzae*, respectively [159]. WRKY TFs also interact with other families of TFs, such as bHLH TFs AtBZR1 and AtBES1/BZR2, working in conjunction with a WRKY TF to regulate BR signaling [160]. Wheat TFs TaWRKY49 and TaWRKY62 have been demonstrated to play roles in resistance to *Puccinia striiformis*, affecting the expression of genes associated with SA, JA, ET, and ROS [161].

Pathogens have evolved effectors that interfere with transcriptional reprogramming by blocking plant TF activity or directly promoting plant gene expression. *P. infestans* effectors prevent potato NAC TFs from moving from the endoplasmic reticulum to the nucleus [162]. *P. syringae* effector HopD1 interacts with the membrane-bound TF NTL9, suppressing effector-triggered immunity [163] and tobacco mosaic virus (TMV) also appears to interfere with the NAC TF ATAF2 to suppress resistance [164]. Pathogens also produce effectors that act as plant TFs [28]. *Xanthomonas* and *Ralstonia* bacteria produce transcriptional activator-like effectors (TALEs) that bind to plant susceptibility gene promoters [23,165]. Rice *Xa10* and pepper *Bs3* genes possess TALE-binding promoter sites for TALEs AvrXa10 and AvrBs3 involved in resistance to *X. oryzae* pv. *oryzae* and *Xanthomonas campestris* pv. *vesicatoria*, respectively [166,167]. While TFs directly trigger transcriptional reprogramming, an additional layer of regulation exists for response-related genes.

### 3.4. Epigenetic Regulation and PDR Provide Additional Regulatory Mechanisms

Nucleic acids have roles as regulators of plant immunity and are critical targets of plant and pathogen degradation. Epigenetic changes can increase pathogen resistance through upregulation of response proteins or downregulation of response inhibitors. Pathogen exposure causes chromatin modification that affects expression of various plant defense response components [168]. DNA methylation, histone methylation/acetylation, RNA interference (RNAi), and recombination between homologous chromosomes influence plant defense [169]. Methylation of genes encoding resistance components has been shown to reduce pathogen resistance [66,170,171]. SA repression activity is facilitated by histone deacetylase HDA19 [172], which interacts with WRKY TFs in response to *P. syringae* [173]. SA analog treatment and *P. syringae* infection of *Arabidopsis* both result in histone acetylation and methylation of gene promoters [168,174]. Epigenetic regulation intensifies the host’s reaction to likely threats, and ensures that host resources are not wasted on resistance to unlikely threats. The physiological cost of defense and longevity of pathogen inoculum both give a selective advantage to plants that can repress and prime responses. Epigenetic factors serve as another layer of regulation of resistance responses by the plant.

In contrast to the ligand–receptor resistance mechanisms discussed earlier, pathogen-derived resistance (PDR) involves the use of pathogen components to confer resistance. Inoculation of a plant with a less virulent form of the pathogen can cause the plant to become more resistant to later infection by a more virulent race/pathotype [175]. Barley primed with chemical elicitors produced progeny with enhanced resistance to the fungi *Rhynchosporium commune* [176]. Interestingly, priming to increase resistance of *Fusarium graminearum* by wheat caused an increase in *F. graminearum* mycotoxin production [177]. PDR to viruses often involves expression of viral coat proteins, replicases, and interfering RNAs [178,179]. Plants expressing the coat protein of a virus resist other viral strains [180] due to interference in viral disassembly that is necessary for viral replication. Viral sequences transcribed by the plant are used to generate microRNAs that interfere with viral transcription, which is necessary for viral replication within the host cell. RNAi is used as an antiviral mechanism that degrades pathogenic RNA. Some pathogens possess effectors that suppress RNA silencing [181,182,183], and RNAi is the underlying mechanism in the development of transgenic wheat with resistance to the wheat streak mosaic virus [184]. RNAi has also been proposed for development of nematode resistance in soybean [185]. Plants regulate defense components using microRNAs, alternative splicing, and alternative polyadenylation [186,187,188,189,190,191,192,193,194]. Pathogens have evolved the ability to use small RNAs as effectors that move into plant cells and repress host defense machinery [195]. In order to facilitate infection, *Botrytis cinerea* uses RNA silencing of the host [196] and *Phytophthora sojae* suppresses RNA silencing [182,197]. Ribonucleases (RNases) in the apoplast have also been correlated with increased resistance to RNA viruses [198] and antifungal activity [199,200,201]. The wheat wheatwin1 protein possesses both RNase and antifungal activity [202]. Corresponding to the diverse receptors and messengers that signal the presence of pests, plants possess a sophisticated array of defense tactics that restrict pathogens and pests from further infection, growth, herbivory, and reproduction.

## 4. Defense Response

### 4.1. HR, ROS, and Cell Wall Modification Inhibit Pathogen Infection

HR is one of the most commonly used immune responses, causing planned cell death in the area surrounding an infection. This establishes a quarantine zone to stop the pathogen from spreading, an effective technique for pathogens that require living tissue (biotrophs). Pathogen infection triggers the production of peroxidases in order to generate ROS, which are used in multiple aspects of the resistance response [203,204]. NADPH oxidases are necessary for the production of superoxide, which peroxidases use to generate hydrogen peroxide (H_2_O_2_). One NADPH oxidase, RBOHD, associates with PRRs EFR and FLS2, and is phosphorylated by BIK1, triggering ROS production [205]. ROS trigger programmed cell death, and hydrogen peroxide moves to surrounding cells to initiate the production of compounds that prevent oxidative damage [206]. Transgenic plants that lack the ability to detoxify ROS compounds were found to have more intense responses to pathogens that trigger HR [207]. Along with assisting in HR, ROS are used to create environments unsuitable for pathogen survival and reproduction, described as an oxidative burst [208]. Therefore, ROS are directly involved at levels of signal transduction and defense response, inhibiting fungal spore germination [208]. In addition to peroxidases and NADPH oxidase, other enzymes produce ROS, including amine and oxalate oxidases [209]. *Sclerotinia sclerotiorum*, a necrotrophic fungus with a broad host range that includes many crops, produces oxalic acid, which suppresses plant oxidative burst [210] in the early stages of infection, but increases ROS production after establishment [211]. Wheat and barley produce oxalate oxidase proteins, also known as germins [212,213], which break down oxalic acid, increasing their pathogen resistance [214]. Transgenic crops with wheat or barley oxalate oxidase genes showed increased resistance to *S. sclerotiorum* [215,216,217] and other pests [218,219,220,221].

ROS mediate glycoprotein crosslinking, which strengthens cell walls [208,222]. Since fungi, bacteria, and nematodes need to penetrate the cell wall, this restricts pathogen movement and limits access to the nutrients necessary to complete reproduction. Bacterial pathogens, lacking some of the degradation enzymes that many fungi possess, use wounds and stomata in order to gain access to plant nutrients. Stomatal guard cells, recognizing bacterial PAMPs (i.e., flg22) and lipopolysaccharides, induce stomatal closure via SA and ABA signaling to prevent entry [137,223]. In response to this, *P. syringae* produces coronatine to initiate reopening of closed stomata [223] by interfering with hormone biosynthesis and mimicking phytohormones [28]. Once viewed as a passive way of pathogen entry into plant tissue, regulation of stomata has been demonstrated to include a complex defense mechanism, in addition to its response to abiotic stress. In addition to oxidase production, plants and pathogens generate many other factors that interfere with each other’s carbohydrates, proteins, and lipids.

### 4.2. Enzymes and Enzyme Inhibitors Counter Pathogenic Effectors and Facilitate Detection

Fungi use enzymes like cellulases to degrade plant cell walls. Upon detection of these fungal proteins, plants respond by producing enzyme inhibitors and depositing callose and lignin to strengthen the cell wall [224]. In addition to cellulases, pathogens like *F. graminearum* and *Fusarium culmorum* degrade plant carbohydrates with pectinases and xylanases [225,226]. In a form of evolutionary retaliation, plants have evolved enzymes that degrade pathogen carbohydrates, including chitinases and β-1-3-glucanases [227,228]. This degradation of fungal cellular components not only inhibits microbial growth, but also makes PAMPs available to plant PRRs, thwarting the pest’s attempt at evasion. Wheat chitinases, degrading a major component of fungal cell walls, inhibit fungal spore germination [229,230]. Recombinant wheat chitinases have been shown to possess antifungal activity against many different fungal species, not limited to wheat pathogenic fungi [231]. The chitin-binding Avr4 of *C. fulvum* protects chitin from plant chitinases [50]. Hevamine possesses chitinase and lysozyme activity and contains β barrel structural domains [232], otherwise associated with a multitude of functions and generally associated with cellular metabolism [233]. Similar to chitinases, plant β-1,3-glucanases hydrolyze β-1,3-glucan in fungal cell walls, producing monomers that further stimulate plant defense responses [234,235]. This multifaceted approach aims at reducing the effectiveness of pathogenic components, as well as strengthening plant defenses. Since the components of cell walls are diverse (i.e., cellulose, hemicellulose, pectin, lignin, etc.), pathogens must have a diverse set of proteins to infect the host, leading to an even more complex arrangement of plant receptors and defense proteins. Thus, the evolutionary battle over the ability to penetrate or reinforce the cell wall is a microcosm for the overall coevolution of the plant–pathogen interaction.

Proteases released by both plants and pathogens evolved to reduce the efficacy of catalytic proteins (i.e., plant chitinase and fungal cellulase). Plants and pathogens also use protease inhibitors to impede the activity of these proteases [236]. Thomas and van der Hoorn discuss ten important types of proteases, grouping them based on location: apoplastic, cytonuclear, vacuolar, endomembrane [237], showing how proteases act at multiple levels in plant–pathogen interaction. The *Fusarium verticillioides* Zn-metalloproteinase fungalysin cleaves defense chitinases [238], but wheat produces hevein-like antimicrobial proteins, that inhibit fungalysin by binding to the enzyme without being cleaved [239], stopping the degradation of chitinases that are necessary to prevent infection. Barley protease inhibitors block the activity of *Fusarium* trypsin, chymotrypsin, and α-amylase [240]. Wheat and barley α-amylase inhibitors interfere with pest starch digestion by interfering with α-amylase, used by insects and fungi to metabolize starches [241,242,243,244,245]. Both proteinaceous and non-proteinaceous α-amylase inhibitors are produced, the latter being organic compounds that mimic α-amylase substrates [245]. *N. attenuata* produces trypsin proteinase inhibitors along with nicotine to defend against *Spodoptera exigua* [246]. Plants may also detect pathogens through protease inhibitors. For example, *C. fulvum*, is perceived by tomato RLP Cf-2 detection of Avr2, which is initially produced to inhibit tomato proteases Rcr3 and PiP1 [247,248]. Oomycete *P. infestans* and nematode *Globodera rostochiensis* interfere with tomato protease activity [249,250], exemplifying a multilayered system of protein–protein interactions. 

Like proteins, lipids participate in many cellular activities. Lipids form major barriers that separate a host plant from prospective pathogens. Pathogens initiate infection after perception of cutin or other compounds of the waxy cuticle. *Puccinia graminis* and *Blumeria graminis* initiate appressoria formation upon contact with surface wax of wheat [251] and barley [252], respectively. Cutinases are then utilized by fungi to hydrolyze cutin into cutin monomers to move through the cuticle [253]. Plant lipases inhibit fungal infection, found in *Arabidopsis* [254] and wheat [255]. Lipid-transfer proteins also possess antimicrobial function linked to increased pathogen membrane permeability. However, details regarding how these proteins affect microbes remain to be elucidated [256]. Effectors that have activity within host cells, such as those of *P. syringae*, require lipid modification to move into the host cell [257], with NLR receptors that detect these effectors located in the plasma membrane instead of the cytoplasm [258,259]. Lipids can also be direct targets of pathogens or plant perception such as the *Fusarium* toxin fumonisin interference with sphingolipid metabolism [260], or defense responses triggered by bacterial lipopolysaccharide PAMPs [261,262]. Phospholipases and lipoxygenases (LOXs) are involved in the breakdown of phospholipids/galactolipids into free fatty acids for the production of defense components [263] and can act in stomatal closure [264]. Phospholipase activity, which is involved with various hormone and stress responses [265], may generate products that are directly involved in defense response, such as phosphatidic acid [263,266]. In addition to this general enzyme activity related to cellular components, plants have evolved specialized proteins that defend them from pathogen infection.

### 4.3. Defensins and Thaumatin-Like Proteins Offensively Inhibit Pathogen Infection

Defensins are a diverse class of small plant proteins that directly attack or inhibit invading microbes and parasitic plants [267]. Initially reported as barley [268] and wheat [269] γ-thionins, it was shown that they possess structural similarity to animal defensins [270,271]. Analogous to the action of many medical antibiotics, plant defensins interfere with pathogen protein synthesis and enzyme function. Barley defensins γ-hordothionin and ω-hordothionin inhibit protein translation [268,272], and defensins can move into pathogen cytoplasm [273,274]. *Triticum aestivum* defensin 1 (Tad1) is expressed in the crown, and possesses antipathogen properties [275]. Defensins inhibit proteases [276], trigger pathogen ROS production [277], and block ion signaling [278]. Unlike animal defensins that inhibit bacterial growth, many plant defensins are antifungal, and are especially active in seeds. They make up 0.5% of the total seed protein and a substantial amount of proteins that are released from the seed coats, at 30% [279]. Defensins have been found in many tissues [280] and may be induced during seasonal changes [275]. C-terminal hydrophobic and γ-core regions are critical for membrane interaction and antifungal activity, respectively [281,282]. Defensins contain scorpion toxin-like, knottin, and purothionin domains, with conserved cysteine residues that form a cysteine-knot structure composed of disulfide bridges. Scorpion toxins and some plant defensins both block potassium channels using similar protein domains [283,284]. Defensins cause an increase in pathogen membrane permeability that initiates necrosis [285]. Previous studies identified over 300 cysteine-rich defensin-like proteins in *Arabidopsis* [286] and *Medicago truncatula* [287]. Van Der Weerden and Anderson have proposed the classification of defensins into 18 groups based on species, structure, and function [288]. Plant defensins have potential as medical antibiotics, antitumor medication [289], and artificial sweeteners [290].

Thaumatin-like proteins, named after the protein thaumatin from *Thaumatococcus daniellii* [291], are also pathogenesis-related proteins. Barley thaumatin-like proteins bind to 1,3-β-D-glucans [292], associated with resistance to powdery mildew [293], *F. graminearum* [294], or general antifungal activity [295]. The antifungal thaumatin-like proteins osmotin (tobacco), zeamatin (maize), hordomatin (barley), avematin (oat), and trimatin (wheat) are permatins that form transmembrane pores in fungal membranes [295,296,297,298]. Like defensins, permatins accumulate in seeds [299]. Research in barley and wheat shows thaumatin-like proteins PRHv-1 and PWIR2, respectively, expressed along with other genes during fungal resistance responses [300,301], with similar sequences in oat [302]. Thaumatin-like proteins have potential as artificial sweeteners and influence the malting quality of barley [303]. Thaumatin-like proteins make up one of the 17 categories of PR proteins: oxidases and oxidase-like (PR-9, 15, and 16), chitinases (PR-3, 4, 8, and 11), β-1,3-glucanases (PR-2), endoproteinases (PR-7), proteinase inhibitors (PR-6), lipid-transfer proteins (PR-14), ribonuclease-like (PR-10), defensins and thionins (PR-12 and 13, respectively), thaumatin-like (PR-5), and the less understood antifungal (Pr-1) and functionally uncategorized (Pr-17) groups [20,304,305,306]. Additional mechanisms that plants use to deter pests involve non-protein compounds and assistance from other species.

### 4.4. Phytoalexins and Beneficial Symbionts Are Chemical and Biological Plant Weapons

Phytoalexins are organic compounds produced in response to invading pests to interfere with metabolism, development, and reproduction. Phytoalexins were initially investigated as defense compounds that protected potatoes from *P. infestans*. Several classes of plant chemicals have pesticide activity and those that are constitutively produced are described as phytoanticipins. A model phytoalexin used by *Arabidopsis*, camalexin, is produced in response to many different types of microbial pathogens and pests [307]. Camalexin is regulated by MAPK cascades [308] and WRKY TFs [155,309,310]. Some adaptive pathogens are able to detoxify this chemical [311]. Wheat produces benzoxazinoids (BXs), such as 2,4-dihydroxy-7-methoxy-1,4-benzoxazin-3-one (DIMBOA), which defends against microbes and insects, such as reducing transmission of barley yellow dwarf virus through aphid feeding [312,313,314]. Wheat pathogens *Gaeumannomyces graminis* and *F. culmorum* can detoxify BXs [315]. Wheat likely inherits genes required to convert 2,4-dihydroxy-1,4-benzoxazin-3-one (DIBOA) to DIMBOA through the progenitor of its B genome (chromosome 4B) since *Aegilops speltoides* accumulates DIMBOA, but *Triticum urartu* and *Aegilops tauschii* do not [316]. Additional phytoalexins in cereals include avenanthramides in oat and diterpenoids in rice [310]. As a phytoanticipin of the saponin group, avenacin A-1 is produced by oat root epidermis and forms pores in fungal membranes by interacting with fungal membrane sterols [317,318,319]. *G. graminis* var. *avenae* has evolved the ability to detoxify avenacin A-1 [320]. While phytoalexins have been found in many species [310] with classified allelopathic effects [321], some signaling mechanisms leading to production remain elusive. Phytoalexins may also show usefulness in medical applications [310,322]. Exogenous chemicals applied to crops, such as glyphosate-based herbicides, may increase crop susceptibility to disease [323].

In addition to producing pesticides, plants can recruit natural predators of herbivorous insects as a defense through the production of herbivore-induced plant volatiles. Caterpillars feeding on maize leaves induce the production of terpenoid compounds and indole, attracting parasitic wasps that feed on the caterpillars [324,325,326]. JA signaling is also activated, driven by volicitin from caterpillar oral secretions [128]. To protect themselves from herbivory, plants can also produce sticky substances that trap insects, such as resin and latex [327,328,329], along with increasing photosynthetic production [330]. Morphological features, like trichomes, reduce insect herbivory, in addition to many cellular components, such as flavonoids, tannins, terpenoids, alkaloids, and phenolics [331]. Aside from providing the plant with access to nutrients, some symbionts assist their host in pathogen defense. Wheat rhizobacteria have activity against the soilborne pathogen *G. graminis* through the production of antibiotic substances [332], and rice arbuscular mycorrhiza trigger improved immune responses to protect the host [333]. Mycorrhizae in corn have the ability to enhance production of DIMBOA [334]. Symbionts have been shown to affect resistance responses through repression of JA-mediated defense [335] or interference with ROS and β-1,3-glucanse production [336,337]. These interactions demonstrate the multilayered nature of the plant immune system.

## 5. Conclusions and Future Directions

Knowledge of plant–pathogen interactions will undoubtedly continue to flourish in the 21st century, driven by new molecular techniques and greater computational power. Phytopathology, like other fields, will continue to grow as more details emerge regarding plant–pathogen interactions. Research will be driven by several factors, such as disease pressures associated with modern agricultural practices and climate change, increasing the need for durable pathogen resistance in crops [338]. In addition to improving our knowledge of plant immunity, efforts will continue to alter crop genetics to develop better resistance. Continuing to alter the receptors necessary to initiate defense responses is likely the best route for development of resistance. NLRs may become a major tool of biotechnology, used to engineer resistance to any pathogen through the modified activity of the CRISPR/Cas9 system. One recent approach utilizes the activation of *Arabidopsis* NLR RPS5 by *P. syringae* protease AvrPphB cleavage of PBS1 [339,340]. Kim et al. showed that the cleavage site of PBS1 can be replaced with a cleavage site for other pathogen proteases, allowing for defense responses to be triggered by other pathogens [341]. While this technology is currently limited, future studies will likely engineer crops with novel R-genes that were not directly transferred from other species. In order to trigger the most effective defense response, engineering novel resistance pathways to different pathogens will also need to pair the receptors with the appropriate method of signal transduction. PDR may also have applications in genetic engineering, by allowing plants to express pathogen genes that promote resistance [342]. Future studies will also focus on understanding quantitative resistance and gene pyramiding, due to the durable resistance they hold [343], such as the multi-decade resistance found in barley cultivar NDB 112 [344,345,346,347]. Additional mechanisms of resistance regulation and response will be uncovered in future years, having applications to agricultural systems. Understanding pathogen resistance and plant immunity will greatly benefit agricultural production by reducing crop loss, and contribute to our understanding of the molecular interactions and coevolution that underlies this field and numerous applications to other biological systems.
